# Antibiotic prophylaxis habits in dental implant surgery among dentists in Spain. A cross-sectional survey

**DOI:** 10.4317/medoral.22626

**Published:** 2018-09-28

**Authors:** Iciar Arteagoitia, Carlos Rodríguez-Andrés, Fabio Rodríguez-Sánchez

**Affiliations:** 1 MD, PhD. Department of Stomatology, School of Medicine and Nursing. University of the Basque Country (UPV/EHU). Barrio Sarriena, s/n, 48940 Bilbao, Spain. BioCruces Health Research Institute member, Cruces University Hospital. Plaza de Cruces, 48903 Barakaldo, Spain; 2 MD, PhD. Professor and Head Epidemiology and Public Health Department, School of Medicine and Nursing. University of the Basque Country (UPV/EHU). Barrio Sarriena, s/n, 48940 Bilbao, Spain; 3 DDS. Epidemiology and Public Health Department, School of Medicine and Nursing. University of the Basque Country (UPV/EHU). Barrio Sarriena, s/n, 48940 Bilbao, Spain

## Abstract

**Background:**

The use of antibiotics to prevent dental implant failures and postoperative infections remains a controversial issue. The objectives of this study were to assess the current antibiotic prescribing patterns and antibiotic prescribing frequency of dentists in Biscay (Spain) in conjunction with routine dental implant surgery among healthy patients and to determine whether any consensus has been reached by such practitioners and last published evidence was being followed.

**Material and Methods:**

Observational cross-sectional study: electronic survey. This study was reported according to the STROBE guidelines. This anonymous questionnaire contained open-ended and close-ended questions. An email was sent 26 October 2017 to all the registered members of the Biscay dentists’ College (n=989). The collected data were analyzed using STATA® 14 software, and 95% confidence intervals (CI) were used to assess the frequency of prescription for each antibiotic regimen.

**Results:**

The survey was responded to by a total of 233 participants (response rate=23.56%). Overall, 210 participants finished the survey completely, and 23 surveys were answered partially. The questionnaire was responded to by 122 females (58.1%) and 88 males (41.9%). Of the participants, 88% (n=207) always routinely prescribed prophylactic antibiotics in conjunction with dental implant surgery (95% CI: 84.79-92.88%). Approximately 9% (n=22) prescribed antibiotics sometimes (95% CI: 5.68-13.19%), and only 4 dentists (1.72%) never prescribed antibiotics (95% CI: 0.04-3.38%). Overall, 179 of 233 respondents prescribed both pre- and postoperative antibiotics (78.85%, 95% CI: 72.96-83.97%), 13 prescribed antibiotics only preoperatively (5.73%, 95% CI: 3.08-9.59%), and 35 prescribed antibiotics exclusively after routine dental implant surgery (15.42%, 95% CI: 10.98-20.78%).

**Conclusions:**

Most of the dentists working in Biscay routinely prescribe prophylactic antibiotics in conjunction with dental implant surgery among healthy patients. A large range of prophylactic regimens are prescribed and the most recently published evidence is not being followed.

** Key words:**Clinical decision making, epidemiology, infection control, dental implants, antibiotics.

## Introduction

Dental implant placement is a routine surgery to replace a lost tooth (1). Despite the fact that dental implants routinely have a high rate of success, dental implant failures occur (2). Bacterial contamination at implant placement might be one of the causes of postoperative infections and early implant failures (3). Infected implants usually have to be removed, and this complication is highly undesirable, both for patients and professionals. For this reason, several prophylactic methods, such as antibiotics, have been used (4).

Nevertheless, the use of antibiotics to prevent dental implant failures and postoperative infections remains a controversial issue (5-7) Unfortunately, there is no consensus among oral health professionals over the use and indications of prophylactic antibiotics in conjunction with dental implant surgeries (8-12).

The use of antibiotics has been the subject of special monitoring since the beginning of the project called European Surveillance of Antimicrobial Consumption (ESAC) in 2001 (13). Spain is actively involved in this project through the Spanish Agency of Medicines and Medical Devices (AEMPS). The latest consumer data on human health, reported in 2016, described Spain as the country with the highest consumption of antibiotics in primary care among the European Union. This high use of antibiotics is also related to a high rate of bacterial resistance. To improve these data, the Spanish Government and the different communities from the Interterritorial Council of the National Health System developed the Spanish Antimicrobial Stewardship Program in Primary Care (PROA) (14). After the implementation of these programs in the hospital, reducing the consumption of antibiotics for 2017-2018 was established as one of the priority objectives. Moreover, a recent review on the antimicrobial prophylaxis in dentistry concluded that antibiotic prophylaxis in healthy patients, for minor oral surgeries, third molar surgeries, implant placement and periodontal surgeries, is not necessary (15).

The use of antibiotics is not indicated in all oral infections, and preventive antibiotics are frequently prescribed to healthy patients (12). The prophylactic use of antibiotics in conjunction with dental implant surgery may be one of these situations (7).

As a result of the present condition, many questions remain, and we asked ourselves what dentists actually do in our province, Biscay: Do they prescribe antibiotics in conjunction with a dental implant surgery? When? Are they following any kind of guidelines? For this reason, we decided to carry out a survey aimed at the total population of registered dentists in Biscay (members of the Colegio Oficial de Dentistas de Bizkaia), a province of the Basque Country in Spain.

The objective of this study is to assess the current antibiotic prescribing frequency and the antibiotic prescribing patterns of dentists in Biscay in conjunction with routine dental implant surgery among healthy patients to determine whether any consensus has been reached by such practitioners and last published evidence is being followed.

## Material and Methods

This observational cross-sectional study was based on an electronic survey approved by the research institute BioCruces (Barakaldo, Biscay). This study was reported according to the Strengthening the Reporting of Observational studies in Epidemiology (STROBE) guidelines (16). Due to the anonymous cross-sectional nature of this study and as it was aimed to professionals instead of real patients, it was granted an exemption in writing by the University of the Basque Country Institutional Review Board (IRB).

-Study Design

A validated questionnaire was prepared to collect information regarding the prescribing patterns of preventive antibiotics among dentists in conjunction with dental implant surgery. The questionnaire followed by Deeb *et al.* was used as a basis, with the explicit permission of the authors (12). The questionnaire has proved its validity, as the different items of the test were found adequate to measure the intended objectives. This anonymous questionnaire comprised data in relation to the following: demographic details, qualification and work experience, most common antibiotic prescribed, duration and dosage. The questionnaire contained both open-ended and close-ended questions ( Table 1,  Table 1 continue,  Table 1 continue-1).

**Table 1:**
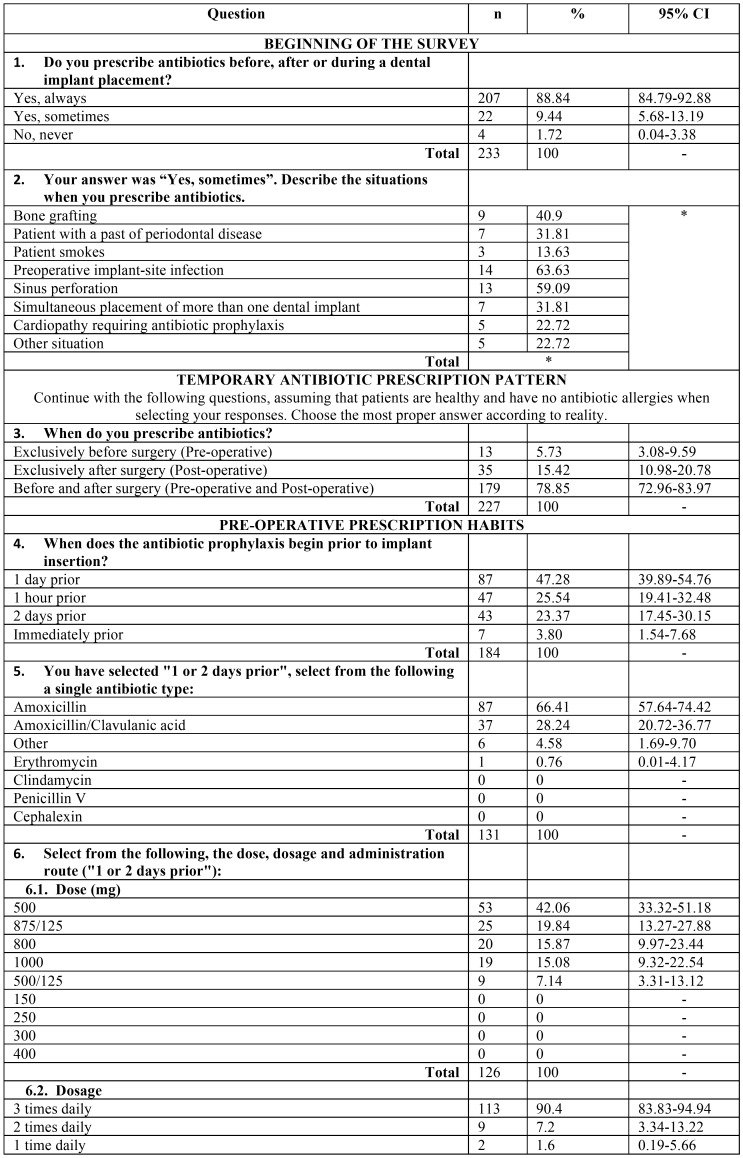
Survey outcome variables: n: Frequency, CI: Confidece Interval, *: respondents could choose more than one option (multianswer).

**Table 1 continue:**
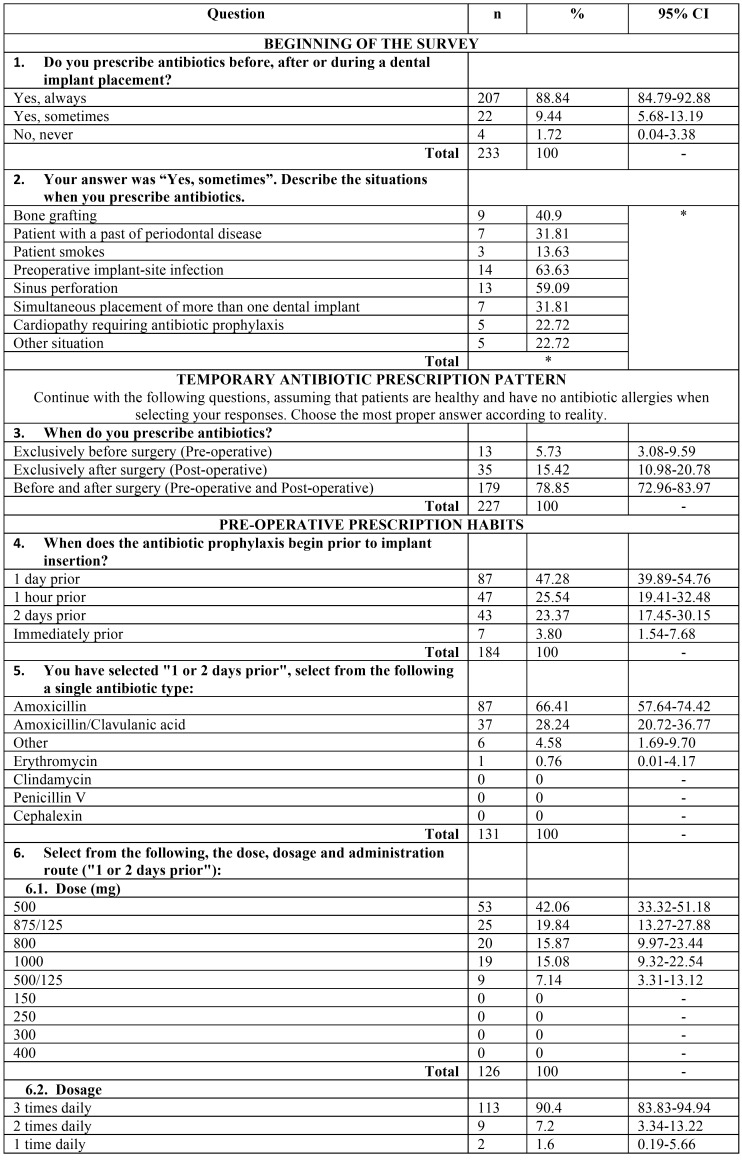
Survey outcome variables: n: Frequency, CI: Confidece Interval, *: respondents could choose more than one option (multianswer).

**Table 1 continue-1:**
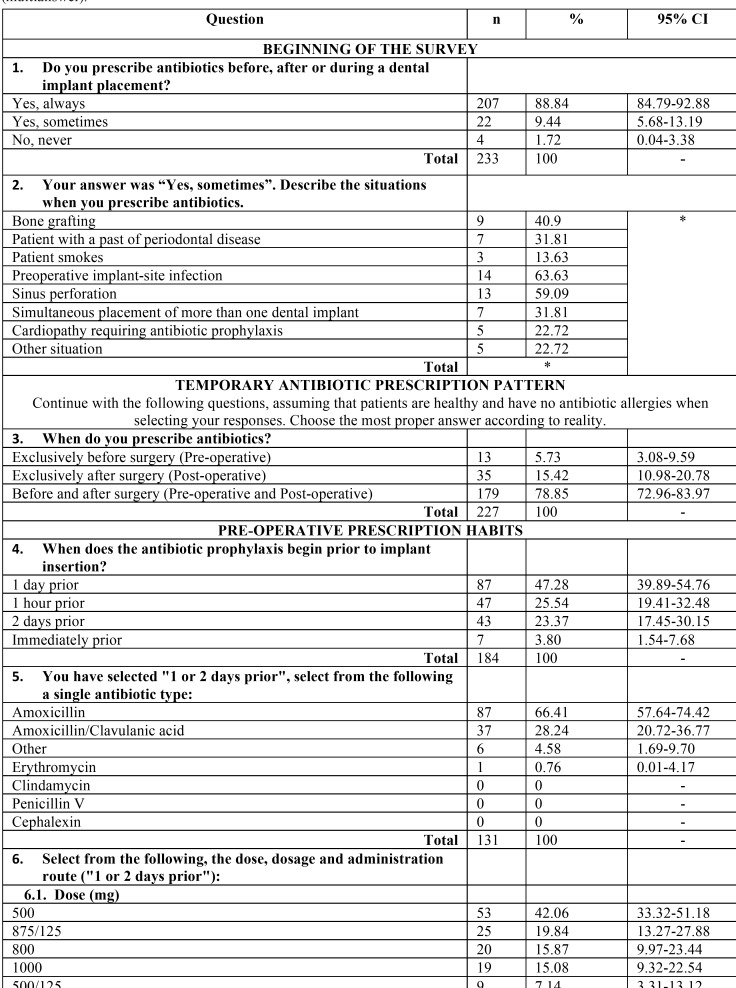
Survey outcome variables: n: Frequency, CI: Confidece Interval, *: respondents could choose more than one option (multianswer).

-Setting

Biscay is a province of Spain located in the Basque Country. Its population was approximately 1,148,302 inhabitants in 2017. An email was sent on 26 October 2017 to dentists including a link to the web questionnaire developed on www.encuestafacil.com. This email also contained instructions to answer the questionnaire if dentists performed dental implant surgeries and a message briefly describing the objectives of the study and the intended use of the collected data for research and epidemiological purposes. It was emphasized that the data were anonymized. A reminder was forwarded on 8 November 2017 to participants who had not responded within the deadline. The online questionnaire was closed to the public on 2 January 2018. Data collection was carried out automatically via the www.encuestafacil.com server.

-Participants

The questionnaire was sent to all the registered members of the Biscay dentists’ College who had not expressly requested not to receive emails. The total number of sent questionnaires was 989. When addressing the entire population of registered dentists of Biscay, the authors understood that the same chance is given (probability) to participate and answer the questionnaire for all members of the association. Participation meant granting the consent of the participant to record the data from the questionnaire.

-Variables

The questionnaire is shown in the  Table 1 with all variables registered.

-Data sources / measurement

Each respondent could only exclusively answer one electronic survey once, and the options for each question are shown in  Table 1.

-Bias

There could not be any selection bias as the electronic survey was sent to all registered dentist in Biscay dentists’ college, and it is mandatory to be registered in one or more dentists’ colleges to work as a dentist in Spain. Similarly, the authors employed an electronic survey previously performed in the United States to avoid information bias.

-Study size

The final sample size comprised the professionals who decided to partially or completely respond the survey (n=233).

-Statistical methods

The collected data were analyzed using Stata 14 software (StataCorp, College Station, Texas, USA); 95% confidence intervals (CI) were used to assess frequency of prescription for each antibiotic regimen.

## Results

-Participants

The survey was responded to by a total number of 233 participants; thus, the response rate was 23.56%. Overall, 210 participants finished the survey completely, and 23 surveys were answered just partially. The descriptive and statistical analyses included all surveys with responses (n=233) to perform as comprehensive an analysis as possible.

-Descriptive data

**Figure 1:**
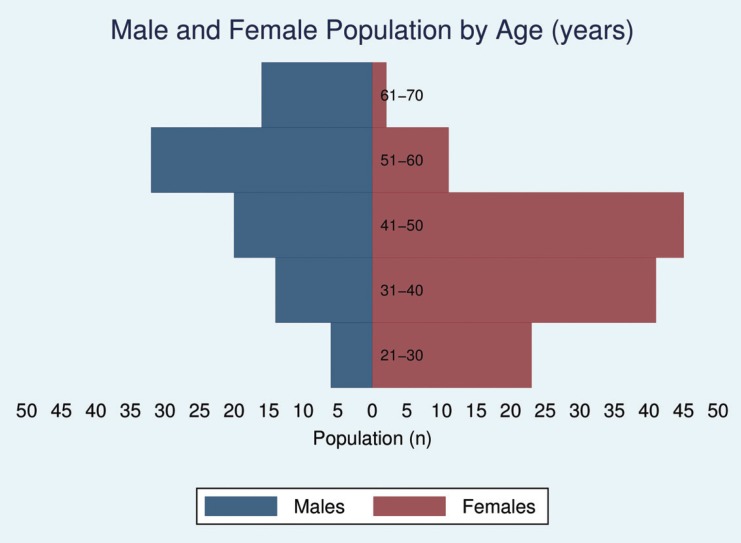
Population Pyramid: No necessary captions.

The questionnaire was responded to by 122 females (58.1%) and 88 males (41.9%), and they were principally aged between 51 and 60 years old (30.95%). A population pyramid is shown in Figure 1.

Overall, 173 respondents had studied in the University of the Basque Country (82.78%) located in Biscay but there were also dentists who had studied at other universities in Spain or in other countries ( Table 1). Approximately 51% of the respondents were working in the rural area of the province, and 43% were working in the capital city of the province, Bilbao. The rest of the respondents were working in another province of Spain (6%).

-Outcome data

 Table 1 shows the percentage of response and the 95% CI for each item. The preoperative and postoperative regimens being followed are shown in  Table 2 and  Table 3.

**Table 2:**
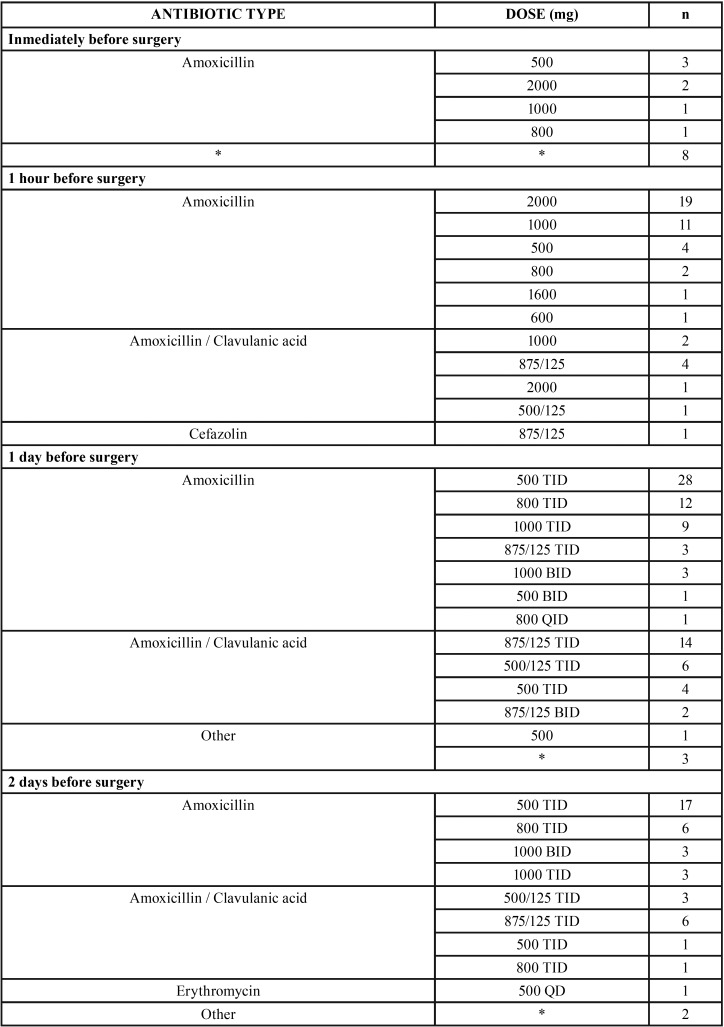
Preoperative regimens: n: frequency, *: the respondents did not answer this question, QD: once a day, BID: twice a day, TID: 3 times daily, QID: 4 times daily.

**Table 3:**
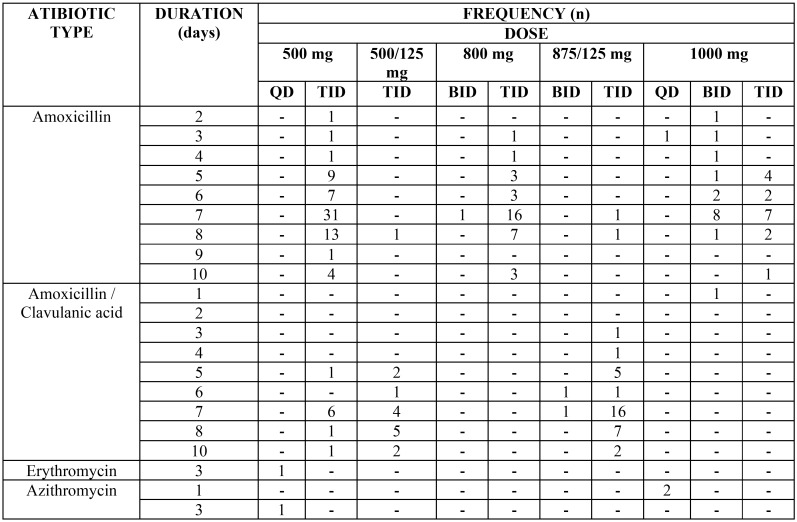
Postoperative regimens: n: frequency of respondents choosing this answer, mg: milligrams, QD: once a day, BID: twice a day, TID: 3 times daily, QID: 4 times daily.

-Main results

**Figure 2:**
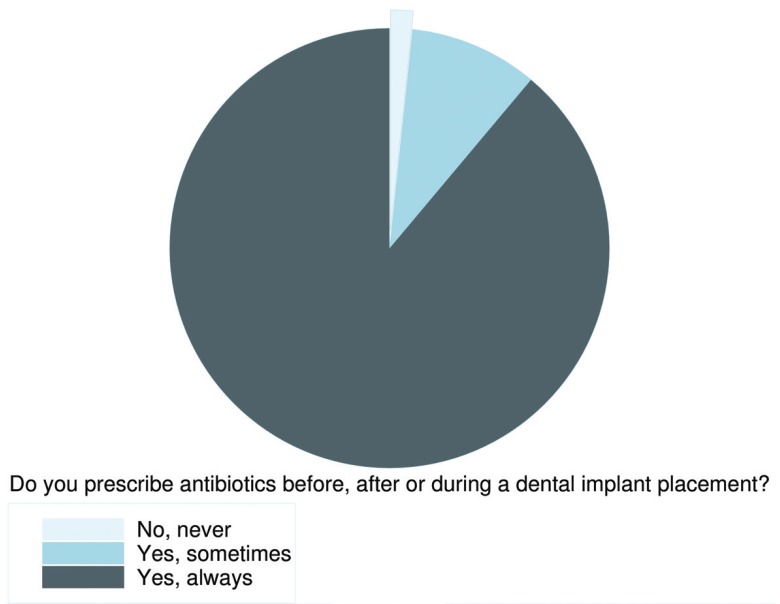
Prophylactic-antibiotics prescription frequency: No necessary captions.

Among all participants, 88% (n=207) always routinely prescribed prophylactic antibiotics in conjunction with a dental implant surgery (95% CI: 84.79-92.88%). Approximately 9% (n=22) prescribed antibiotics sometimes (95% CI: 5.68-13.19%), and only 4 dentists (1.72%) did not prescribe antibiotics at all (95% CI: 0.04-3.38%), (Fig. 2).

The 22 dentists prescribing antibiotics only “sometimes” were asked to determine the situations when they do prescribe them. The most-often selected conditions were a preoperative implant-site infection (n=14) and sinus perforation (n=13).

**Figure 3:**
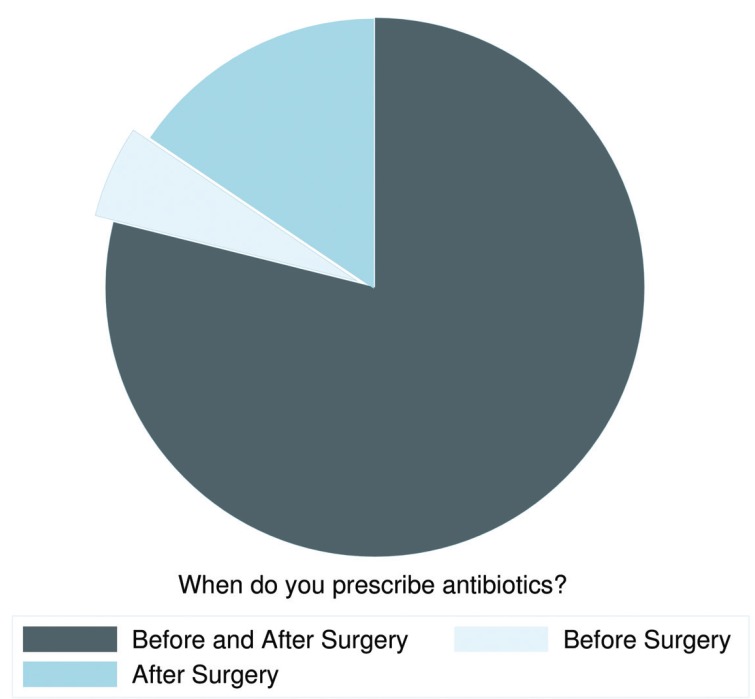
Time patterns of antibiotics prescription: No necessary captions.

Overall, 179 of 233 respondents prescribed both pre- and postoperative antibiotics (78.85%, 95% CI: 72.96-83.97%), 13 prescribed antibiotics only preoperatively (5.73%, 95% CI: 3.08-9.59%), and 35 prescribed antibiotics exclusively after a routine dental implant surgery (15.42%, 95% CI: 10.98-20.78%), (Fig. 3).

The only route of administration described by all respondents was orally for all antibiotic types and regimens.

Of the 179 respondents who indicated that they prescribed preoperative and postoperative antibiotics, the most common preoperative regimen was 500 mg amoxicillin three times a day (TID) 1 day before surgery (n=25), and the most frequent postoperative regimen was 500 mg amoxicillin TID orally for 7 days after surgery (n=24). This pre- and postoperative regimen was consistently followed by a total of 10 dentists.

Of the 13 respondents who prescribed exclusively preoperative antibiotics, the most common antibiotic regimen was 2 g amoxicillin once orally 1 hour before surgery (n=3) and 500 mg amoxicillin TID 1 day before surgery (n=3).

Of the 35 dentists who exclusively prescribed postoperative antibiotics, the most frequent regimen was 500 mg amoxicillin TID for 7 days after surgery (n=7).

After amoxicillin, amoxicillin/clavulanic acid was the most routinely prescribed antibiotic type. The most frequent prescription with amoxicillin/clavulanic acid was pre- and postoperative (n=49). Among the respondents who followed this pre- and postoperative prescription pattern, the most common preoperative regimen was 875/125 mg TID 1 day before surgery (n=14), and the most frequent postoperative regimen was 875/125 mg TID orally for 7 days after surgery (n=16).

## Discussion

-Key results 

Most dentists in Biscay routinely prescribe prophylactic antibiotics in conjunction with dental implant surgery. Approximately 88% of the respondents always prescribed prophylactic antibiotics. A large range of prophylactic regimens is followed, which demonstrates the substantial variety of choices made by dentists. This fact also reveals a lack of consensus among professionals. In addition, recommendations made in the last published evidence are not being followed.

The review performed by Exposito *et al.* suggested that a single dose of 2 or 3 g amoxicillin 1 hour preoperatively significantly reduces dental implant failures (2). Moreover, the review conducted by Rodríguez Sánchez *et al.* concluded that postoperative amoxicillin (associated or not with preoperative amoxicillin) might not be beneficial preventing dental implant failures and postoperative infections (7). Therefore, the prescription habits of most of the dentists might be considered overtreatment, as approximately 93% of the respondents prescribed some kind of postoperative regimen after surgery. Furthermore, only 3 dentists (1.28%) exclusively prescribed 2 g amoxicillin 1 hour before surgery.

-Limitations 

This survey was sent to all dentists registered in Biscay, and it was focused on the antibiotic prescription patterns involving dental implant surgery among health patients without any allergies.

To assure the confidence of the respondents, the authors and institution were precisely introduced, and the objectives were explained in the email with the link. This introduction message sent with the link to the questionnaire stated that the survey should only be answered by dentists performing dental implant surgeries. However, the electronic survey could have been responded to by professionals who do not perform dental implant surgeries. Unfortunately, there is no possible way to avoid and control this fact, but this number is assumed to be low.

It is also not possible to know the total number of dentists who perform dental implant surgeries in Biscay; however, the percentage of response seems to be coherent with the professionals carrying out dental implant surgeries in the province.

The survey could only be answered exclusively by a single respondent. The authors consider the response percentage high. Nevertheless, there are some aspects that cannot be controlled, such as the reliability and authenticity of the answers obtained.

-Interpretation

The lack of standardized protocols may be the reason for the routine and systematic prescription of antibiotic regimens without enough scientific evidence supporting a beneficial effect (especially for postoperative regimens) (2,7). The current situation might increase the incidence of the adverse effects associated with the overtreatment with antibiotics, such as bacterial resistance and other systemic disorders. Therefore, this condition must be considered of interest to the dentistry community and national health systems because of epidemiological and economic reasons.

This fact has encouraged the development of many other surveys in the last years performed in very diverse countries, such as Jordan, the United Kingdom (UK), Sweden, and the United States (US) (8,9,10,12).

Although there is limited evidence supporting antibiotic prophylaxis, the high percentage of professionals in other countries prescribing prophylactic antibiotics routinely in conjunction with a dental implant placement is remarkable. The prescription percentages among professionals range from almost 50% to 75% among the respondents in these other studies. However, in Biscay this statistic reaches 88%.

The results suggested by the present survey of dentists from Biscay are similar to those reported by other authors, who also found great variation in antibiotic prescribing regimens with respect to types, dose and treatment duration. Amoxicillin was found to be the most frequently prescribed antibiotic type in other survey studies, except in Jordan, which favors amoxicillin with clavulanic acid. Similar to the habits in Biscay, the practice of prescribing amoxicillin 2 g preoperatively was also found in Sweden and the US as the most common preoperative regimen. On the other hand, professionals from the UK and the US were aligned on the most frequently prescribed postoperative regimen, as they prescribed 500 mg amoxicillin 3 times daily for 5 days. However, dentists from Biscay lengthen this postoperative prescription pattern until 7 days after surgery.

-Generalizability

This survey was carried out among respondents registered as dentists in the College of Biscay, and most of them had studied at the University of the Basque Country (UPV/EHU). Consequently, other professionals who had been trained in other institutions in Spain may generate other patterns of prescription regarding the use of prophylactic antibiotics in dental implant surgeries. Nevertheless, the systematic use of prophylactic antibiotics among dentists and the large variation in the prescription regimens being used, most of them based on no scientific evidence, may be generalized.

In conclusion, dentists in Biscay routinely use prophylactic antibiotic in conjunction with a dental implant placement among healthy patients. A large range of prophylactic regimens is followed, and high variability among the prescribed regimens is shown. However, a consecutive pre- and postoperative regimen with amoxicillin is the most frequently prescribed.

Unfortunately, recommendations made by the most recent published evidence are not being followed. Protocols and guidelines are needed to define the indications for prophylactic antibiotic prescription in dental implant surgery to avoid overtreatment with antibiotics and the associated risks and economic costs.
